# Supplementary material for: A comprehensive survey and comparative analysis of time series data augmentation in medical wearable computing
Source: PLoS One. 2025 Mar 18;20(3):e0315343. doi: 10.1371/journal.pone.0315343 (PMC11957733; doi:10.1371/journal.pone.0315343)
Supplement: S1 Table — (PDF) [file pone.0315343.s002.pdf]

S1 Table: Generator hyper-parameters

**OPPORTUNITY**		**HAR**		**DEAP**		**PMDB & BVDB**	
Layer Type	Output Shape	Layer Type	Output Shape	Layer Type	Output Shape	Layer Type	Output Shape
G latent input layer	(None, 100)	G latent input layer	(None, 100)	G latent input layer	(None, 100)	G latent input layer	(None, 100) & (None, 100)
G label input layer	(None, 1)	G label input layer	(None, 1)	G label input layer	(None, 1)	G label input layer	(None, 1) & (None, 1)
G Combined layer	(None, 8, 107, 1)	G Combined layer	(None, 16, 9, 17)	G Combined layer	(None, 8, 40, 3)	G Combined layer	(None, 44, 1, 3) & (None, 80, 1, 3)
conv2DTranspose_1	(None, 16, 107, 64)	conv2DTranspose_1	(None, 32, 9, 64)	conv2DTranspose_1	(None, 16, 40, 64)	conv2DTranspose_1	(None, 88, 1, 64) & (None, 160, 1, 64)
Activation	LeakyRelu	Activation	LeakyRelu	Activation	LeakyRelu	Activation	LeakyRelu
Dropout	0.20	Dropout	0.30	Dropout	0.20	Dropout	0.40
conv2DTranspose_2	(None, 32, 107, 64)	conv2DTranspose_2	(None, 64, 9, 64)	conv2DTranspose_2	(None, 32, 40, 64)	conv2DTranspose_2	(None, 176, 1, 64) & (None, 320, 1, 64)
Activation	LeakyRelu	Activation	LeakyRelu	Activation	LeakyRelu	Activation	LeakyRelu
Dropout	0.20	Dropout	0.30	Dropout	0.20	Dropout	0.40
Conv2D	(None, 32, 107, 1)	conv2DTranspose_3	(None, 128, 9, 64)	conv2DTranspose_3	(None, 64, 40, 1)	conv2DTranspose_3	(None, 352, 107, 1) & (None, 640, 1, 64)
Activation	tanh	Activation	LeakyRelu	Activation	LeakyRelu	Activation	LeakyRelu
		Dropout	0.30	Dropout	0.20	Dropout	0.40
		Conv2D	(None, 128, 9, 1)	conv2DTranspose_4	(None, 128, 40, 32)	conv2DTranspose_4	(None, 704, 1, 32) & (None, 1280, 1, 64)
		Activation	tanh	Activation	LeakyRelu	Activation	LeakyRelu
				Dropout	0.20	Dropout	0.40
				Conv2D	(None, 128, 40, 1)	conv2DTranspose_5	(None, 1408, 1, 32) & (None, 2560, 1, 64)
				Activation	tanh	Activation	LeakyRelu
						Dropout	0.40
						Conv2D	(None, 1408, 1, 1) & (None, 2560, 1, 1)
						Activation	tanh
