# Supplementary material for: A comprehensive survey and comparative analysis of time series data augmentation in medical wearable computing
Source: PLoS One. 2025 Mar 18;20(3):e0315343. doi: 10.1371/journal.pone.0315343 (PMC11957733; doi:10.1371/journal.pone.0315343)
Supplement: S2 Table — (PDF) [file pone.0315343.s003.pdf]

# S2 Table: Discriminator hyper-parameters

| **OPPORTUNITY** | | **HAR** | | **DEAP** | | **PMDB & BVDB** | |
|---|---|---|---|---|---|---|---|
| **Layer Type** | **Output Shape** | **Layer Type** | **Output Shape** | **Layer Type** | **Output Shape** | **Layer Type** | **Output Shape** |
| D Label input layer | (None,1) | D Label input layer | (None,1) | D Label input layer | (None,1) | D Label input layer | (None,1) & (None,1) |
| G input layer | (None,32,107,1) | G input layer | (None,128,9,1) | G input layer | (None,128,40,1) | G input layer | (None,1408,1,1) & (None,2560,1,1) |
| D Combined layer | (None,32,107,2) | D Combined layer | (None,128,9,2) | D Combined layer | (None,128,40,2) | D Combined layer | (None,1408,1,2) & (None,2560,1,2) |
| conv2D_1 | (None, 16,54,64) | conv2D_1 | (None,128,9,64) | conv2D_1 | (None, 64,40,64) | conv2D_1 | (None,704,1,64) & (None,1280,1,64) |
| Activation | LeakyRelu | Activation | LeakyRelu | Activation | LeakyRelu | Activation | LeakyRelu |
| conv2D_2 | (None,8,27,128) | Dropout | 0.80 | Avg_pooling | (None,32,40,64) | Avg_pooling | (None,352,1,64) & (None,640,1,64) |
| Activation | LeakyRelu | Conv2D_2 | (None,128,9,64) | conv2D_2 | (None,16,40,128) | conv2D_2 | (None,176,1,128) & (None,320,1,64) |
| Maxpooling2D | (None, 3,13,128) | Activation | LeakyRelu | Activation | LeakyRelu | Activation | LeakyRelu |
| Flatten | (None, 4992) | Dropout | 0.80 | Avg_pooling | (None,8,40,128) | Avg_pooling | (None,88,1,128) & (None,160,1,64) |
| Dropout | 0.2 | Flatten | (None, 73728) | Flatten | (None,40960) | Flatten | (None,11264) & (None,20480) |
| Dense_1 | (None, 512) | Dropout | 0.40 | Dense_1 | (None, 128) | Dense_1 | (None,128) & (None,128) |
| Dense_2 | (None, 256) | Dense_1 | (None, 16) | Dense_2 | (None, 64) | Dense_2 | (None,64) & (None,64) |
| Dense_3 | (None, 64) | Dense_2 | (None, 1) | D_output_layer | (None, 1) | D_output_layer | (None,1) & (None,1) |
| D_output_layer | (None, 1) | Activation | Sigmoid | Activation | Sigmoid | Activation | Sigmoid |
| Activation | Sigmoid | Optimizer | SGD(lr=0.0002) | Optimizer | SGD(lr=0.0001) | Optimizer | SGD(lr=0.0001) |
| Optimizer | Adam (lr = 0.0002) | Loss | Binary_crossentropy | Loss | Binary_crossentropy | Loss | Binary_crossentropy |
