# Supplementary material for: A comprehensive survey and comparative analysis of time series data augmentation in medical wearable computing
Source: PLoS One. 2025 Mar 18;20(3):e0315343. doi: 10.1371/journal.pone.0315343 (PMC11957733; doi:10.1371/journal.pone.0315343)
Supplement: S3 Table — (PDF) [file pone.0315343.s004.pdf]

S3 Table: Average accuracy scores of the DA approaches on OPPORTUNITY. The baseline average accuracy without augmentation is 89.41%. Lighter colours indicate better results.

| factor \ Method | Jitter | Rotation | Scaling | MW | Slicing | TW | WW | PRM | RGW | DGW | SPAWNER | cGAN |
|---|---|---|---|---|---|---|---|---|---|---|---|---|
| 0.2 | 88.91 | 89.59 | 89.77 | 89.04 | 89.97 | 89.43 | 89.99 | 89.79 | 89.74 | 89.89 | 89.59 | 89.66 |
| 0.4 | 89.86 | 89.94 | 89.73 | 89.36 | 89.53 | 89.94 | 90.26 | 89.85 | 89.79 | 89.94 | 89.74 | 89.60 |
| 0.6 | 89.31 | 89.49 | 89.99 | 89.94 | 89.93 | 89.62 | 89.74 | 89.83 | 89.82 | 89.80 | 89.77 | 89.35 |
| 0.8 | 89.98 | 89.58 | 89.54 | 89.83 | 89.50 | 89.88 | 89.67 | 89.46 | 89.49 | 89.72 | 89.68 | 89.55 |
| 1 | 89.42 | 89.49 | 89.53 | 89.32 | 89.23 | 89.49 | 89.44 | 89.45 | 89.66 | 89.30 | 88.79 | 89.15 |
| 2 | 89.14 | 89.36 | 89.73 | 89.72 | 89.79 | 89.80 | 89.65 | 89.23 | 89.41 | 89.41 | 89.29 | 89.61 |
| 3 | 89.51 | 89.21 | 89.21 | 89.91 | 89.28 | 89.56 | 89.38 | 89.53 | 89.70 | 89.94 | 88.62 | 89.25 |
| 4 | 89.58 | 89.31 | 89.86 | 89.42 | 89.63 | 89.96 | 89.83 | 89.49 | 89.52 | 89.78 | 89.28 | 89.26 |
| 5 | 89.59 | 89.47 | 89.48 | 89.92 | 89.74 | 90.03 | 89.83 | 89.23 | 89.54 | 89.65 | 89.59 | 88.82 |
| 6 | 89.76 | 89.01 | 89.83 | 89.61 | 90.04 | 89.78 | 89.41 | 89.21 | 90.06 | 89.96 | 89.14 | 89.10 |
| 7 | 90.08 | 88.76 | 89.93 | 89.69 | 89.98 | 90.25 | 90.20 | 89.66 | 89.94 | 89.92 | 89.26 | 89.20 |
| 8 | 89.87 | 89.19 | 89.52 | 89.90 | 89.79 | 89.76 | 89.88 | 89.31 | 90.14 | 89.99 | 89.51 | 88.85 |
| 9 | 89.75 | 89.25 | 89.97 | 89.57 | 90.21 | 90.77 | 89.78 | 89.84 | 89.96 | 89.90 | 89.29 | 89.20 |
| 10 | 89.43 | 89.36 | 89.88 | 89.92 | 89.92 | 89.78 | 89.31 | 89.55 | 90.12 | 89.68 | 89.11 | 88.71 |
