# Supplementary material for: A comprehensive survey and comparative analysis of time series data augmentation in medical wearable computing
Source: PLoS One. 2025 Mar 18;20(3):e0315343. doi: 10.1371/journal.pone.0315343 (PMC11957733; doi:10.1371/journal.pone.0315343)
Supplement: S4 Table — (PDF) [file pone.0315343.s005.pdf]

S4 Table: Average accuracy scores of the DA approaches on HAR. The baseline average accuracy without augmentation is 91.56%.

Factor \ Method	Jitter	Rotation	Scaling	MW	Slicing	TW	WW	PRM	RGW	DGW	SPAWNER	GAN
0.2	92.94	93.53	92.76	93.42	92.32	92.90	92.26	94.82	93.33	92.98	92.87	92.87
0.4	94.05	92.56	92.93	92.68	93.6	92.31	92.93	94.78	92.90	94.08	92.35	89.78
0.6	92.15	90.74	93.28	93.7	92.61	92.54	92.72	93.97	92.87	94.16	92.21	93.09
0.8	92.69	90.62	93.51	93.03	92.20	92.49	93.00	94.54	93.06	93.51	92.82	91.68
1	92.48	93.70	93.80	92.80	93.27	95.34	93.61	94.68	93.02	92.77	93.01	92.08
2	92.51	89.58	94.12	93.40	92.30	94.41	92.35	94.44	93.53	92.79	91.89	88.51
3	92.46	70.33	93.27	93.16	92.70	94.73	93.75	93.65	93.56	91.82	92.27	89.07
4	91.96	58.90	93.01	93.13	91.25	94.60	92.62	93.63	92.94	92.16	87.93	84.76
