# Supplementary material for: A comprehensive survey and comparative analysis of time series data augmentation in medical wearable computing
Source: PLoS One. 2025 Mar 18;20(3):e0315343. doi: 10.1371/journal.pone.0315343 (PMC11957733; doi:10.1371/journal.pone.0315343)
Supplement: S7 Table — (PDF) [file pone.0315343.s008.pdf]

S7 Table: Accuracy scores of the DA approaches on BVDB. The baseline avg. accuracy without DA is 83.71%

Factor \ Method	Jitter	Rotation	Scaling	MW	Slicing	TW	WW	PRM	RGW	DGW	SPAWNER	cGAN
0.2	83.89	82.19	83.75	83.68	83.78	83.89	83.63	83.84	83.75	83.64	83.78	83.71
0.4	83.63	80.98	83.74	83.47	83.49	83.96	83.69	83.80	83.40	83.50	83.75	83.61
0.6	83.28	80.59	83.64	83.48	83.45	83.97	83.40	83.72	83.31	83.22	83.71	83.62
0.8	83.32	80.20	83.87	83.45	83.48	83.59	83.63	83.60	83.27	83.21	83.66	83.65
1	83.44	80.17	83.79	83.63	83.60	83.97	83.45	83.67	83.36	83.08	84.00	83.53
2	82.83	79.41	83.64	83.50	83.46	83.92	84.98	84.34	83.97	83.48	83.80	83.57
3	83.00	79.40	83.69	83.42	83.43	84.20	85.39	84.44	84.18	83.02	83.96	83.61
4	82.51	79.54	83.84	83.19	83.26	84.43	85.90	84.58	84.32	82.88	84.08	83.59
