# Supplementary material for: A comprehensive survey and comparative analysis of time series data augmentation in medical wearable computing
Source: PLoS One. 2025 Mar 18;20(3):e0315343. doi: 10.1371/journal.pone.0315343 (PMC11957733; doi:10.1371/journal.pone.0315343)
Supplement: Attachment — Submitted filename: Response_to_Reviewers.pdf [file pone.0315343.s010.pdf]

Response to Reviewers' Comments:

Reviewer 1:

We would first like to thank you for your comprehensive feedback! The latter has been really helpful and allowed us to identify weaknesses in the current version of our manuscript. We answered all your comments as follows:

Comments	***Our Response***
1. The topic is interesting, however for a full-fledged survey paper I would say the coverage is not sufficient. This is rather an experimental study as they have done several experimentation to understand the impact of data augmentation.	Our initial intention when writing this paper was to propose a work in the middle between a survey and an experimental study. The main reason behind this decision is that although several fairly comprehensive surveys on the topic of time series data augmentation have been recently published, they all lack a comprehensive experimental application of the surveyed augmentation techniques to various types of time series datasets (e.g. univariate and multivariate, balanced or unbalanced in the case of labeled datasets, etc.). We identified this shortcoming as a research gap that we wanted to fill with our paper. We nevertheless take from your comment the idea that the survey part of our paper might not have been developed enough relatively to its experimental part. We therefore added Section 2 - Related Work that provides a more detailed analysis of recent TSDA works from the recent literature.
2. The challenges in the said field should be discussed elaborately. Also discuss the motivations behind the work, shading some lights on the data augmentation aspect as one of the research challenges.	Based on your feedback, we enriched Section 1.2 with more detailed information regarding the challenges of TSDA. An analysis of the related literature as well as our own experience with time series data processing led us to identify three main challenges that are specific to TSDA (as opposed to augmentation with other modalities such as images):<br><br>1- Data scarcity: in practice, it is quite costly both in time and resources to acquire TS datasets, especially for medical-related applications. This leads to the available datasets to be relatively small. TS data is also scattered in the sense that merging datasets coming from different sensors or devices is not a trivial process. All these factor contribute to large amounts of TS data not to be available, which is an obstacle to the development of generative methods (e.g. cGANs) that require large amounts of training data. This issue can be exacerbated by class imbalances in the case of labelled datasets, since some classes may be underrepresented and associated to less data, which makes training a model to generate samples for this class even more challenging.<br><br>2- Label integrity: TS data can be sensitive to small modifications that change its semantic meaning, which can cause issues for

	augmentation approaches which assume that augmented example retains the same label as the original sample it is coming from.<br><br>3- Difficulty of the assessment of the generated data quality: unlike images whose semantic content can usually easily be visually assessed, it can be difficult to ensure that the generated TS data still contains meaningful content.<br><br>These challenges are currently still not addressed and actively investigated in the TSDA literature. Consequently, most of the approaches proposed so far either bypass data scarcity issues by not requiring a data learning-based process or hinge on the assumption that small modifications to the original TS preserve its class label and semantic content. We added a reference to all aforementioned elements in Section 1.2.
3. In the research gap section, can they be more specific on the gaps that previous surveys did not address/cover?	Time series data augmentation (TSDA) is a topic that has recently attracted some notable interest from the research community due to its relevance to many application domains, especially those connected to ubiquitous computing. As a result, many publications concerning this topic have been proposed in the past few years. But these papers all have their shortcomings that can be summarised along the following main axes:<br><br>1- The vast majority of the literature investigates data augmentation (DA) for univariate time series only, which is a strong limitation since data acquired from wearable sensors in ubiquitous computing applications are usually multivariate.<br><br>2- Many past works are simply surveying the field for TSDA without providing concrete results obtained in an experimental analysis. Works that include experimental results also tend to ignore more computationally expensive approaches like generative models such as GANs.<br><br>3- Past related works usually do not provide practical recommendations regarding the application of TSDA.<br><br>4- The related literature that includes experimental results does not investigate data augmentation on medical-related data, which is an important application field for TSDA.<br><br>We modified Section 1.3 so that it incorporates a reference to the four aforementioned contributions of our study compared to the existing work.
4. Section 2.1: They should discuss more clearly the reasons for proposing such taxonomy.	The taxonomy proposed in this paper is a filtered version of the one introduced in Iwana et al. [1] that is adjusted to take into account DA approaches applicable to multivariate TS data, as Iwana's study was tested exclusively on univariate data. We added information

	related to this aspect to Section 3.1 (previously 2.1).<br><br>[1] B. Iwana et al., *An empirical survey of data augmentation for time series classification with neural networks*, Plos One, 2021
5. Can they write the captions for Figs. 2-3 in a more descriptive way to understand the differences among different data augmentation methods.	To incorporate your suggestion into our manuscript, we added details about how each DA approach of the RT family of augmentations modifies the original signal with a reference to each method parameters in the caption of Figure 2. When it comes to Figure 3 that shows examples of DA techniques from the PM family, the interpretation is less trivial. RGW, DTW and SPAWNER are all based on aligning the time series to be augmented to another reference time series using Dynamic Time Warping (DTW), with the main differences between the three approaches residing in the selection of the reference, or the variant of DTW that is applied. We therefore added sentences referring to the aforementioned elements in the caption of Figure 3.
6. I suggest the authors to use more datasets.	Following your suggestion, we performed additional experiments using the Human Activity Recognition Using Smartphones (HAR) dataset, a widely used benchmark dataset for HAR introduced by Anguita et al. [2]. A description of the dataset and the results of the experiments carried out on it were introduced in Section 4.1.1 and 5.1 respectively.<br><br>[2] D. Anguita et al., *A Public Domain Dataset for Human Activity Recognition using Smartphones*, Proceedings of ESANN 2013
7. I suggest the authors to use different GAN models.	To address your comment, we considered two potential alternatives: regular GANs and Wasserstein GANs. Unfortunately, both options had drawbacks that led us not to include them in the experiments presented in this paper. The main issue with regular and Wasserstein GANs is the fact that they are both models trained in an unsupervised manner. In order to produce labeled augmented data, training one GAN per class would therefore be required. This is suboptimal in our case for two reasons: 1- some of the datasets we used (e.g. OPPORTUNITY) contain many classes which would lead to many models to train; 2- most datasets we used have a relatively small amount of data per class, which could lead to difficulties in training each class-specific model properly. In our experiments, we observed overall that generative-based approaches required lots of effort to properly train and underperformed relatively to the other tested data augmentation approaches. This led us to think that an additional set of experiments specifically focused on generative approaches (e.g. GAN-based, diffusion models) that investigate parameters such as training schemes and architectures would be required to improve the performances of this type of approach. We considered this to be too much for the experiments presented in this paper, and therefore decided to leave

	this as future work. A mention to the aforementioned points was added in the Conclusion section.
8. They need to improve Section 5 significantly to understand the impact of the data augmentation issues.	As a preliminary remark, we would like to mention that the additional experiments we carried out in the revision process slightly modified the results we reported in the previous version of our manuscript. The performances of the previously best-performing overall approaches (TW and WW) were further improved, while the relative ranking of the other approaches remains unchanged. More specifically, cGANs still poorly perform on all tested datasets. This is probably due to the fact that RT approaches such as TW and WW are currently the most suitable to deal with the challenges of TSDA: since they are not learning-based methods, they are less affected by data scarcity issues. It is also hypothesised that the changes they introduce in the original TS are modest enough to preserve the label and semantic content integrity.<br><br>Following these observations, we added further comments describing the obtained results on a general level in the Discussion section (Section 7, previously Section 5). More specifically, we added Table 9 that shows the rank of all tested approaches based on their average F1 scores on all five datasets and updated the contents of Sections 7.1 and 7.2. We also added a Section 7.3 to discuss the practical challenges we faced in using generative augmentation approaches (i.e. cGANs).
9. As they are considering this as a survey paper, they need to discuss the limitations and future research avenues more strongly.	We consider that the main limitation for multivariate TSDA is highlighted by the subpar performances obtained by generative models as evidenced in our experimental results. This is hypothesised to be due to challenge #1 that we mentioned in Section 1.2, i.e. the scarce amount of available TS data that prevents a proper training of complex models such as cGANs. We believe that potential for future studies resides in refining deep-learning-based approaches for TSDA, for instance by performing a more in-depth exploration of more elaborate model architectures that would be more adapted to small datasets. Additionally, it would be good to check the generalisation capacity of our results on multivariate TS datasets that are not related to medical applications. We added a reference to the aforementioned elements in Sections 7.3 and 8 of our manuscript.
10. There are some typographical and grammatical errors. Please reread the paper and correct those.	We performed an additional spell check and corrected the grammatical errors we found in our manuscript.

Reviewer 2:

First of all, we wanted to thank you for your detailed and comprehensive feedback! Your comments were helpful to us in identifying the weaknesses of the current version of our manuscript. More specifically, we addressed each of them as follows:

Comments	*Our Response*
1. The metrics and parameters being used are not consistent, which makes comparisons difficult (Accuracy and F1-Score)	Both accuracy and average F1 scores were originally computed on all datasets. Because of concerns about the space it would take, we decided not to present both in our manuscript, and made the choice to show only the accuracy/F1-score for balanced/unbalanced datasets, respectively. In the light of your comment however, we understand that this can lead to confusion for the reader. We therefore decided to present only the average F1-score for all datasets in the manuscript, and moved reports of the accuracy in the Appendix (Tables 14 to 19).
2. Maybe it could be helpful to provide results for all augmentation factors for all experiments (e.g. 0.2, 0.4, 0.6, 0.8 for "Opportunity" and 3-10 for the others) and F1-scores for all datasets	Following your comment, we added the results of the data augmentation techniques for factors smaller than 1 on the OPPORTUNITY dataset, which can be seen in Table 4. Larger augmentation factors however proved to be more problematic for some datasets (e.g. DEAP in particular) due to the amount of data becoming too large to train the classification models in a practical manner. As a compromise, we added experiments including augmentation factors up to 4 for the other datasets. The updated results including the new augmentation factors on all datasets are reported in Tables 4 to 9 for the average F1 score, and Tables 14 to 19 in the Appendix for the accuracy.
3. Moreover, it is insufficient to report mean values only, instead an appropriate measure of dispersion should be considered as well (maybe other than variance, due to the small number of samples). Something like a Critical Difference Diagram would be helpful in judging the results of the experiments (Friedman test and post-hoc Nemenyi). Simple classifiers for a baseline comparison	Based on your suggestion, we performed a Friedmann test and post-hoc Nemenyi and reported their results in the newly added Section 6. The Friedmann test confirmed that there were significant differences (p-value<=0.05) in the performances obtained by all DA approaches and the baseline on all tested datasets. In Figure 8, we added the matrices of p-values for the post-hoc Nemenyi carried out on all datasets. These results reveal that the performance of the best DA approach is always significantly different from the one of the baseline, although it may not be significantly different from some other DA methods. A comment on the results of this experiment was added to Section 6. One additional remark: we also plotted Critical Difference Diagrams according to your suggestion, that we are enclosing to this response letter (c.f. The attached Figure). The results unfortunately looked messy and not so easy to interpret to us, which is why we decided to leave them out of the manuscript. We hypothesise this is because our sample size to carry the test is relatively small (n=5 scores obtained for each DA approach), which negatively affects the precision of the confidence intervals. Increasing this sample size unfortunately proved to be quite

could be helpful (something like multivariate dynamic time warping classifiers)	difficult, as this incurred a very significant increase in computational time (expected additional computational time to double n from 5 to 10: 2-4 months). This is mostly explained by the high costs of the cGAN-based methods, and the fact that each DA method must be applied again n times, in a 5-fold-cross-validation setup, on all 5 datasets, for a large number of augmentation factors. We think performing more repetitions would become more realistic in a study focusing on a lower number of DA techniques, especially if generative approaches are left out.<br><br>For the same argument of keeping computational times reasonable, we also decided to leave your suggestion of testing additional classifiers as future work. We added a mention to this in the Conclusion section.
4. Finally, it would be interesting to get more information about the connection between dataset characteristics and DA methods. As an example: "Rotation" impairs performance on BVDB, PMDB and Opportunity but improves performance for emotion recognition. How does this relate to the characteristics of DEAP and what general conclusion may be drawn from this?	As a preliminary remark: the interpretation of why the “rotation” augmentation improves performances on some datasets is not trivial, since “rotation” (whose term was coined in Iwana et al. [1]) is an operation that is an element-wise scaling of the time series elements with multiplicative factors randomly taken in [-1,1], rather than a mathematical rotation that would be easier to interpret. Moreover, the additional experiments carried out to revise the manuscript slightly modified the results we previously reported. The performances of the best DA techniques overall such as TW and WW were further improved, while the ones of rotation relatively degraded (which led us to remove Figure 9 from our previous manuscript).<br><br>Performing a low-level analysis of the obtained results proved to be non-trivial, so we decided to enrich the Discussion Section (Section 7, previously 5) with elements discussing our experimental results on a high level. More specifically, we added Table 9 that summarises the ranks obtained by the tested DA approaches and the baseline based on their average F1 scores on all five datasets and updated the previous content of the section. We also added Section 7.3 to discuss the practical challenges we faced when applying generative augmentation approaches.<br><br>[1] B. Iwana et al., *An empirical survey of data augmentation for time series classification with neural networks*, Plos One, 2021
5. Check the reproducibility of the result from the GitHub Code	We checked again that the code could be properly executed to reproduce the results reported in our paper. A README file containing instructions on how to use the code was also added to make its usage easier.

Additional remarks:

**Remarks**	**Our Response**
1. Equations should be embedded into sentences (e.g. equation (1)).	We checked again Section 3 (previously Section 2) to make sure all Equations were properly embedded into the text.
2. Matrices (e.g. equation (2)) could be typeset using \vdots, \ddots, etc.	The matrices in Section 3 (previously Section 2) were reformatted according to your comment.
3. There is a typo in equation (14).	The typo in Equation (14) was corrected.
4. The naming of the variable "macro F_1 score" could be reconsidered. Using words in equations could be avoided.	References to the “macro F1 score” were changed to “average F1 score” and abbreviated to “AF1”. The equations in Section 4.5 were modified to use brief notations instead of full words.
5. There seems to be a mistake in the first sentence of Section 3.2.	The first sentence of Section 4.2 (previously 3.2) was corrected.
6. The colors in Fig. 8 are unnecessary and might be misleading.	Based on your suggestion, we removed the color scaling in this Figure (now Figure 9 after the modifications to the manuscript).
7. The y-axis in Fig. 8 could be limited to a smaller range (e.g. 50 to 90) in order to make the results more recognisable.	The Y axis of Figure 9 was modified to display a smaller range.
8. The notation in equation (10) and (11) could be improved.	The min and max notations in Equations 10 and 11 were improved.
9. Something seems to be missing in the second sentence of Section 6.	The second sentence of this Section (now Section 8 after revisions) was rewritten.

10. It could be helpful to make a difference in notation of matrices and vectors and scalars.	To make the differences between scalars, vectors and matrices more obvious, we used the following convention: bold font and lower case for vectors, bold font and upper case for matrices, regular font for scalars.
11. The parameters that appear in Table 3 are not sufficiently documented. For example \Omega is introduced as a random matrix in the context of Jittering (equation (3)). In table 3, however, it seems that \Omega denotes variance in the context of scaling. As an example, it might be more instructive to write something like $\alpha \sim \mathcal{N}(0.1,1)$ in row 3 of table 3.	Your comment made us realise that Table 3 contained erroneous information more specifically for scaling, MW and TW, likely due to some wrong copy/paste operation. We apologize for this oversight, and corrected the table by adding the appropriate information.
12. The meaning of \mathbb{R}^{+*} in Section 3.4 (line 460) is unclear.	$\mathbb{R}^{+*}$ designates the set of real numbers that are strictly positive (+: positive; *: non zero), but this is a notation that may not be standardised. We therefore modified it for disambiguation purposes to $]0,+\infty[$ in the manuscript.
13. The axis labels and legends in almost all figures are not readable in a print version (Figs. 2,3,4,5,7,8,9,10,11).	We revised the Figures mentioned in your comment by either increasing their size or modifying the axes labels to increase their readability.
14. The formal descriptions of DA methods in section 2 should be reconsidered. E.g. :<br>- It is unclear in how far equation (3) describes a rotation. The components of R hold random values between -1 and 1. The Hadamard product of this matrix and a data matrix X doesn't seem to realize a rotation.	- As described in one of our previous answers, the term "rotation" was originally introduced by Iwana et al. [1]. We re-used their Python implementation for our experiments and realised that "rotation" (as they implemented it) does not refer to an actual mathematical rotation, but rather an element-wise scaling of the time series elements with multiplicative factors randomly taken in [-1,1] (controlled by cos(theta) with theta~[0,2*Pi[). We also believe that this name is misleading for this reason, but decided to keep it to preserve the consistency between our and Iwana's taxonomies. To clarify the aforementioned elements, we added sentences to describe them in Section 3.2.1.

- What is the meaning of \mathsf{T} in the exponent in equation (5)? There seems to be no need for transposition.<br>- equation (9) is inconsistent in notation.	- The T symbol in Equation 5 indeed refers to the transpose symbol. We added it because MW performs the scaling on the rows $x^{(i)}$ of X (i.e. on a timestamp-level basis), and from our understanding, $x^{(i)}$ in the notation $X = (x^{(1)}, …, x^{(n)})$ usually designates the columns of X. We added a reminder to the dimensionality of $x^{(i)}$ after Equation 5 so that it is easier to understand the purpose of the transposition.<br><br>- The inconsistency in Equation 9 mostly came from an incorrect definition of x and x_{nn}. We modified the description of SMOTE as well as Equation 9 in Section 3.3.1 to make both clearer overall.<br><br>[1] B. Iwana et al., *An empirical survey of data augmentation for time series classification with neural networks*, Plos One, 2021

Journal Requirements:

1. Please ensure that your manuscript meets PLOS ONE's style requirements	We thoroughly checked the images, table, file naming conventions, authors name, and affiliation in the manuscript, and modified them to fit the requirements of PLOS ONE.
2. Role of funders	We updated the role of funders as you suggested in the acknowledgment section in the article.
3. Availability of dataset	In our study, we used 5 datasets (OPPORTUNITY, HAR, DEAP, BVDB, and PMDB). Except for PMDB, all the others are publicly available online. A publication presenting and describing the PMDB dataset was submitted to *Scientific Data* (Nature Portfolio) in July this year, with the second round of reviews completed on 28.08.2024. Pending the publication of this article, we provide the coordinates of Prof. Dr.-Ing. Marcin Grzegorzek as the contact point of the institution responsible for the dataset:<br><br>Research Group: Medical Data Science<br>Institute Name: Institute of Medical Informatics<br>Contact person: Professor Dr.-Ing. habil. Marcin Grzegorzek<br>Location: Building 64, 2nd floor, Room 89, University of Luebeck, Germany<br>Website:<br>https://www.imi.uni-luebeck.de/forschung/ag-medical-data-science
4. Remove supplementary files from the main manuscript.	We also removed the supplementary figures and tables from the main manuscript. We submitted them separately in a separate file called "Supplementary Materials"

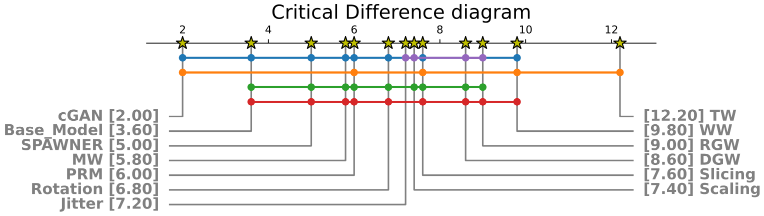

(a) **OPPORTUNITY**

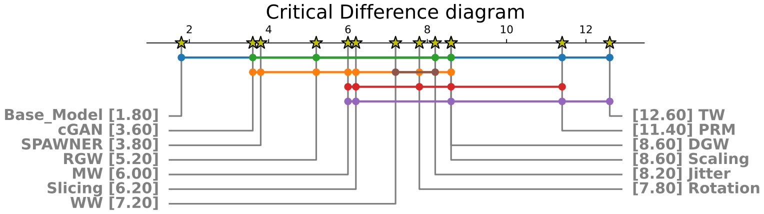

(b) **HAR**

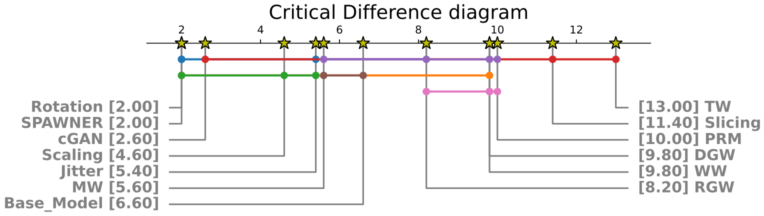

(c) **DEAP (Arousal)**

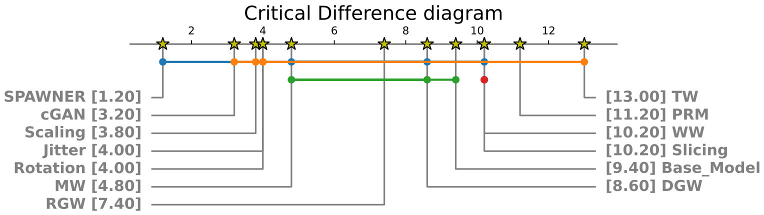

(d) **DEAP (Valence)**

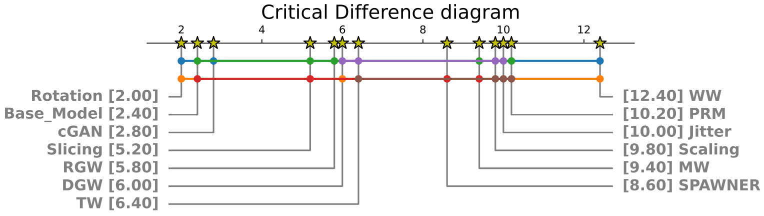

(e) **PMDB**

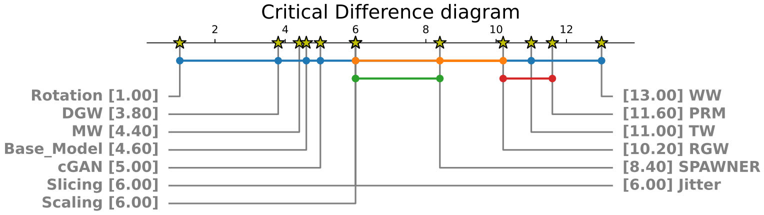

(f) **BVDB**

Figure A: **Critical difference plot based on the Nemenyi posthoc test across all performance measures**
