# Supplementary material for: A comprehensive survey and comparative analysis of time series data augmentation in medical wearable computing
Source: PLoS One. 2025 Mar 18;20(3):e0315343. doi: 10.1371/journal.pone.0315343 (PMC11957733; doi:10.1371/journal.pone.0315343)
Supplement: Attachment — Submitted filename: Response_to_reviewers_auresp_2.pdf [file pone.0315343.s011.pdf]

Revision 2

Response to the Reviewer's Comments:

We are thanking once again the reviewer for providing us with their detailed feedback! We are happy to learn that most of the previously raised points have been considered as properly addressed. We very carefully considered your remaining points, and are providing our answer to them below.

Comments	***Our response***
Parts of the statistical analysis are unclear (to me). For example: Is Figure 8 reporting on pair-wise significance tests? This would be unusual (probability for random validation grows with number of methods, see for example [Janez Demsar, 2006]).	Figure 8 is indeed reporting the pair-wise significance tests between all tested DA approaches on all datasets, as the critical difference diagrams we obtained ended up being somewhat difficult to interpret. As mentioned in our previous answer, the number of repetitions of our experiments (n=5) was kept low due to the high computational cost caused by the large number of tested parameters and DA techniques to train (especially for generative DA methods). We think it leads to the assumption enounced by J. Demsar in the introduction of his paper [1] to be unfulfilled (c.f. “We will only assume that the measured results are ‘reliable’; to that end, we require that enough experiments were done on each dataset [...]”). Our number of runs is in particular lower than the ones normally used in related machine learning studies we found that are also applying the post-hoc Nemenyi, such as Fawaz et al. [2] (n=10), Ma et al. [3] (n=100), etc. Increasing this number would be more feasible in a study where fewer configurations are tested, i.e. by focusing on specific DA methods, datasets and/or augmentation factors for instance. We added details related to the aforementioned information in Section 7.3.<br><br>Your comment about the probability of random validation increasing with the number of tested approaches is very relevant, and made us realise we overlooked this factor in our previous analysis. 13 methods compared to each other in our case means c=12*13/2=78 pair-wise comparisons, which means a family-wise error rate (FWER) of around 0.98. To perform an adjustment, we applied the Bonferroni

	correction that corrects the significance-level by a factor 1/c, i.e. an updated significance of 0.0135/78=0.00064. We updated the tables of pairwise comparisons in Figure 8 with this new significance level. These new results do not change the overall conclusions we drew from our previous analysis, i.e. that the overall performances of the DA approaches are dataset-dependent although random transformations tend to perform better, and that the top performing approaches on each dataset may not yield statistically significantly different results from the next best methods, but consistently beat the baseline model. We updated Section 6 to explain the aforementioned changes.<br><br>References:<br>[1] J. Demsar, Statistical Comparisons of Classifiers over Multiple Data Sets, Journal of Machine Learning Research, 2006<br>[2] H. I. Fawaz et al., Deep learning for time series classification: a review, Data Mining and Knowledge Discovery, 2019<br>[3] J. Ma et al., A comprehensive comparison among metaheuristics (MHs) for geohazard modeling using machine learning: Insights from a case study of landslide displacement prediction, Engineering Applications of Artificial Intelligence, 2022
There are still some minor issues in notation, type setting and typography. Notation and type setting are flawed especially regarding the punctuation of equations. Some captions are still not recognisable in a print version (e.g. Fig 11).	Following your advice, we performed an additional check of our manuscript which allowed us to correct some minor residual grammatical mistakes, and improve some mathematical notations, especially in Section 3.<br><br>We additionally modified Figures 7, 9 and 11 to make the text and captions easier to read, by either removing redundant text, increasing the Figure size, or reorganizing the Figure contents.
